# *Bartonella rochalimae* and Other *Bartonella* spp. in Fleas, Chile

**DOI:** 10.3201/eid1507.081570

**Published:** 2009-07

**Authors:** Laura Pérez-Martínez, José M. Venzal, Daniel González-Acuña, Aránzazu Portillo, José R. Blanco, José A. Oteo

**Affiliations:** Hospital San Pedro-Centro de Investigación Biomédica de La Rioja, Logroño, Spain (L. Pérez-Martínez, A. Portillo, J.R. Blanco, J.A. Oteo); Universidad de la República, Montevideo, Uruguay (J.M. Venzal); and Universidad de Concepción, Chillán, Chile (D. González-Acuña)

**Keywords:** Bartonella rochalimae, Pulex irritans, Chile, Bartonella henselae, Bartonella clarridgeiae, Ctenocephalides felis, dogs, cats, bacteria, letter

**To the Editor:** Fleas are involved in the natural cycle of different *Bartonella* spp. Among the 20 currently recognized *Bartonella* spp., 13 species or subspecies have been implicated in human disease. Recently, *B. rochalimae* was identified in a patient who had received numerous insect bites and subsequently had bacteremia, fever, and splenomegaly after visiting Peru (*1*). A recent study in Taiwan suggested that rodents could be a reservoir for *B. rochalimae* (*2*), but the vector or other mechanism of infection remains unknown. We amplified *B. rochalimae*, *B. clarridgeiae*, and *B. henselae* from fleas (*Pulex irritans* and *Ctenocephalides felis*) collected in Chile and discuss the role of these fleas as possible vectors of infection.

From 2005 through 2008, we collected 82 fleas from cats and dogs in pounds in Chile: 34 *P. irritans*, 37 *C. felis,* and 11 *C. canis*. Fleas were kept in 70% ethanol and sent to the Special Pathogens Laboratory of the Área de Enfermedades Infecciosas of the Hospital San Pedro, La Rioja, Spain, to be examined for *Bartonella* spp. Fleas were then rinsed in distilled water and dried on sterile filter paper under a laminar-flow hood. Each flea was crushed with a sterile pestle, and DNA was extracted by lysis with 0.7 M ammonium hydroxide. PCR was used to detect *Bartonella* DNA (according to the defining criteria for *Bartonella* spp.); primers targeted the RNA polymerase β-subunit–encoding gene (*rpoB*) and the citrate synthase gene (*gltA*) (*3*–*5*). PCR primers for a fragment of the 16/23S rRNA intergenic region and the heat-shock protein-encoding gene (*groEL*) were also used (*6*,*7*). Positive controls (*B. henselae* strain Marseille, kindly supplied by Unité des Rickettsies, Faculté de Médecine, Université de la Méditerranée, Marseille, France) and negative controls (sterile water instead of template DNA) were used. PCR products were purified, and both strands of each amplicon were subjected to sequence analysis. Nucleotide sequence homologies were searched by using BLAST (www.ncbi.nlm.nih.gov/blast/Blast.cgi).

When *rpoB* primers were used, *Bartonella* spp. were found in 4 *C. felis* (4.8%) fleas from cats and in 4 *P. irritans* (4.8%) fleas from dogs. The same 8 samples were positive when primers for *gltA* gene and 16/23S rRNA intergenic region were used. Unfortunately, none of the 82 specimens were positive when PCR primers targeting the *groEL* gene were used. In all experiments, negative controls remained negative.

Sequencing of the 825-bp *rpoB* fragments from the 4 *C. felis* fleas indicated that they were most closely aligned with the gene sequences of *B. clarridgeiae* (n = 2, >99% similarity) and *B. henselae* (n = 2, 100% similarity). Using *gltA* (380 bp), we found 100% similarity with *B. clarridgeiae* and *B. henselae*. Accordingly, the 16/23S rRNA amplicons from these specimens exhibited 100% similarity with the corresponding sequences of *B. clarridgeiae* (154 bp) *and B. henselae* (172 bp).

Amplicons for the *rpoB* fragment gene obtained from 4 *P. irritans* fleas showed highest similarity (97.2%–99.5%) with *rpoB* of *B. rochalimae.* Three were identical, and we deposited the consensus sequence in GenBank in 2006 under the name “uncultured *Bartonella* sp.” and accession no. DQ858956. The sequence differed from those described for all known *Bartonella* spp. and phylogenetically was most closely related to *B. clarridgeiae* (*8*). The sequence of the protein encoded by *rpoB* in these 3 specimens (protein_id ABH09235) had 3 aa changes (121I→V, 233K→I, and 274N→E) with respect to the deduced sequence of the RpoB protein for *B. rochalimae*. The importance of these changes remains unknown. The remaining nucleotide sequence was recently submitted to GenBank under accession no. FJ147196, designated *B. rochalimae* because isolation of this new *Bartonella* spp. was reported in 2007 (*1*). These 4 specimens also yielded positive PCR products for *gltA* (380 bp) and 16/23S rRNA (≈175 bp). Subsequent nucleotide sequence analysis showed 100% homology with the corresponding partial nucleotide sequences from *B. rochalimae*.

In 2002, Parola et al. (*9*) amplified *Bartonella* DNA by using PCR with *Pulex* spp. fleas collected from persons in Peru and suggested the existence of a new *Bartonella* sp. The nucleotide sequence of the 16S-23S ribosomal RNA intergenic spacer obtained from 1 genotype (clone F17688) was nearly identical to the corresponding sequence of *B. rochalimae*. This finding suggests that *Pulex* spp. fleas could be vectors.

Cat scratch disease has been reported in Chile, and *B. henselae* has been found in cats in Chile (*10*). Thus, our finding of *B. henselae* and *B. clarridgeiae* in *C. felis* fleas from Chile confirms the risk for exposure of humans in contact with cat fleas. Furthermore, our finding of *B. rochalimae* in *P. irritans* fleas from dogs in Chile supports the possibility that *P. irritans* fleas could be vectors for *B*. *rochalimae*. These findings are of public health importance because they identify possible vectors of these human pathogens.
